# Compression Therapy in Patients Aged 65 and Older with Late Stage II Secondary Lower Limb Lymphedema

**DOI:** 10.3400/avd.oa.26-00030

**Published:** 2026-08-14

**Authors:** Midori Tsukagoshi, Jiro Maegawa

**Affiliations:** 1 Faculty of Nursing, School of Medicine, Tokai University, Isehara, Kanagawa, Japan; 2 Department of Plastic and Reconstruction Surgery, Yokohama City University School of Medicine, Yokohama, Kanagawa, Japan

**Keywords:** compression therapy, compression stockings, grip strength, lower limb lymphedema, older patients

## Abstract

**Objectives:**

This study aimed to clarify the relationship between selection of compression stockings and grip strength in older outpatients with secondary lower limb lymphedema following gynecologic cancer surgery.

**Methods:**

This retrospective observational study involved 71 outpatients aged ≥65 years with late-stage II (International Society of Lymphology) secondary lower limb lymphedema requiring compression therapy after gynecologic cancer surgery. We investigated the patient’s profile, grip strength, and the status of compression therapy. Patients were divided into early-older (65–74 years; n = 41) and late-older (75–86 years; n = 30) groups, and outcomes were compared. Additionally, patients were divided by compression stocking types: a flat-knit stocking group (n = 51) and a circular-knit stocking group (n = 20).

**Results:**

All patients continued compression therapy during the observation period between September 2022 and April 2025. Grip strength was comparable between the early-older and late-older patient groups. However, the flat-knit stocking group had significantly greater grip strength (21.1 kg) than the circular-knit stocking group (19.1 kg) (*p* = 0.004).

**Conclusions:**

Even late-older patients could continue to use flat-knit stockings as long as their grip strength was maintained.

## Introduction

In recent years, Japan has become the world’s most rapidly aging country, with 29.1% of its total population aged ≥65 years, 16.1% aged ≥75 years, and 10.1% aged ≥80 years.^1)^ The number of patients with gynecologic cancer is increasing in Japan,^2)^ and consequently, the number of older patients with secondary lower limb lymphedema after cancer treatment is expected to rise.

The standard treatment for secondary lymphedema is complex decongestive therapy (CDT), which primarily involves long-term compression therapy.^3–5)^ Additionally, limb volume reportedly decreases with exercise under compression during the perioperative period of lymphaticovenous anastomosis (LVA), a surgical treatment for lymphedema.^6–9)^ Unlike regular stockings, the compression stockings used in CDT exert high compression pressure; accordingly, it is necessary to acquire the required skills and maintain a certain level of muscle strength to don them on and doff them. However, as skeletal muscle mass decreases with age, grip strength—which reflects upper limb strength—peaks at 40–49 years of age and then gradually declines.^10)^ Consequently, as patients get older, it becomes more difficult for them to don and doff the compression stockings used to treat lymphedema.

At our plastic surgery outpatient clinic, we treat many patients with severe secondary lymphedema who wear high-stiffness, flat-knit compression stockings. Many older patients are worried about not being able to continue compression therapy. In clinical settings, compression stockings must be tailored to each patient, and individuals should receive appropriate instruction on their use to ensure long-term adherence based on physical function.^11)^

In some countries, local healthcare professionals collaborate to support patients with daily compression garment application and removal.^12)^ Previous studies have reported the effectiveness of compression instruction using multilayer bandaging techniques.^13)^

However, in Japan, such support systems are rare, there is a shortage of caregivers, and patients are generally required to manage their compression stockings independently or rely on assistance from family members.

This study aimed to investigate the relationship between selection of compression stockings and grip strength in patients aged ≥65 years with late-stage II secondary lower limb lymphedema (International Society of Lymphology) following gynecologic cancer surgery, and to clarify trends across age groups.

## Materials and Methods

### Study design and patient population

This retrospective observational study included female patients aged ≥65 years diagnosed with secondary lower limb lymphedema following gynecologic cancer surgery. Patients with primary lower extremity lymphedema—cases of stage 0, stage I, and early stage II, according to the International Society of Lymphedema (ISL)—were excluded.

### Data collection

Out of 188 women who visited the outpatient plastic surgery departments of 2 hospitals between September 2022 and April 2025, the data of 83 women aged ≥65 years with lower limb lymphedema were initially analyzed. Of these, 7 patients were excluded: 2 with primary lower limb lymphedema and 5 with milder disease not requiring compression therapy. Seventy-six patients were in the late stage of stage II lymphedema. Furthermore, 1 patient who used Class III flat-knit stockings and 4 patients who used Class I stockings were excluded. Finally, 71 patients who used Class II stockings were diagnosed with late-stage II lymphedema. Patients were classified into 2 age groups based on the Japanese classification of older adults: 65–74 years (early-older, Group A) and 75–86 years (late-older, Group B).

Data on lymphedema severity, manual lymph drainage (MLD), walking duration in a day while wearing compression stockings, grip strength, types of compression stockings, and any other clinical data were collected from patients’ medical records. Grip strength was measured using a digital handheld dynamometer (Grip-D; Takei Scientific Instruments, Niigata, Japan), and the higher value of the 2 measurements taken from either hand was used for analysis. Furthermore, patients were grouped based on the types of compression stockings used (flat-knit vs. circular-knit) for comparative analysis.

### Statistical analyses

The Mann–Whitney *U* test was used to assess differences in age and grip strength between the 2 age groups (Groups A and B) and to assess differences in grip strength between the circular-knit and flat-knit compression stocking groups. All analyses were performed using SPSS version 28 (IBM, Armonk, NY, USA), with a significance level of *p* <0.05.

## Results

### Patients' profile

Patient data are summarized in **Table 1**. Lymphedema developed following surgery for cervical cancer in 42 patients, uterine cancer in 21, and ovarian cancer in 8; lymph node dissection was performed in all the patients. Unilateral lymphedema occurred in 19 patients in Group A and 30 in Group B, while bilateral lymphedema occurred in 12 patients in Group A and 10 in Group B. It has been more than 10 years for all the patients since they were diagnosed with secondary lower limb lymphedema. The median Body Mass Index (BMI) of Group A was 23.7 (range: 19.9–31.3) kg/m^2^ and that of Group B was 23.0 (range: 16.1–29.4) kg/m^2^, with no significant difference between the 2 groups. None of the patients continued MLD; furthermore, the patients wore compression stockings and walked around their neighborhoods every day, but this was not as intense as exercise. All patients lived with their families—none lived alone—and they were able to don and doff the compression stockings themselves without assistance.

**Table 1:** Patient’s profile (n = 71)

Variable	Group A (n = 41) 65–74 years	Group B (n = 30) 75–86 years	*p*-Value
Age (years)	70	78	0.001*
Body Mass Index (BMI: kg/m^2^)	23.7 (19.9–31.3)	23.0 (16.1–29.4)	0.22 (n.s)
Types of gynecologic cancers and the number of patients			
Cervical cancer	24	18	
Uterine cancer	13	8	
Ovarian cancer	4	4	

*Mann–Whitney *U* test (*p* <0.05).

n.s, no significance

### Types of compression stockings

Types of compression stockings that were worn by the patients included circular-knit compression stockings (Class II: 23–32 mmHg) and flat-knit compression stockings (Class II: 23–32 mmHg). No patients used elastic bandages. In Group A, 32 (78.0%) patients used flat-knit compression stockings and 9 (21.9%) used circular-knit compression stockings. In Group B, 19 (63.3%) patients used flat-knit compression stockings and 11 (36.7%) used circular-knit compression stockings. **Table 2** shows the results for 71 patients wearing circular-knit and flat-knit compression stockings. Most patients put on the compression stockings using rubber gloves and sliders, whereas none of the patients used frame-type supportive devices. The changes in the volume for the patients during the observation periods were within plus or minus 10%.

**Table 2:** Types of compression stockings used in each group (n = 71)

	Types of compression stockings			
	Number of patients	Flat-knit compression stockings class II	Circular-knit compression stockings class II	*p*-Value
Group A 65–74 years	41	32 (78.0%)	9 (21.9%)	0.221 (n.s)
Group B 75–86 years	30	19 (63.3%)	11 (36.7%)	

Mann–Whitney *U* test (*p* <0.05).

n.s, no significance

### Grip strength (Fig. 1)

**Fig. 1:**
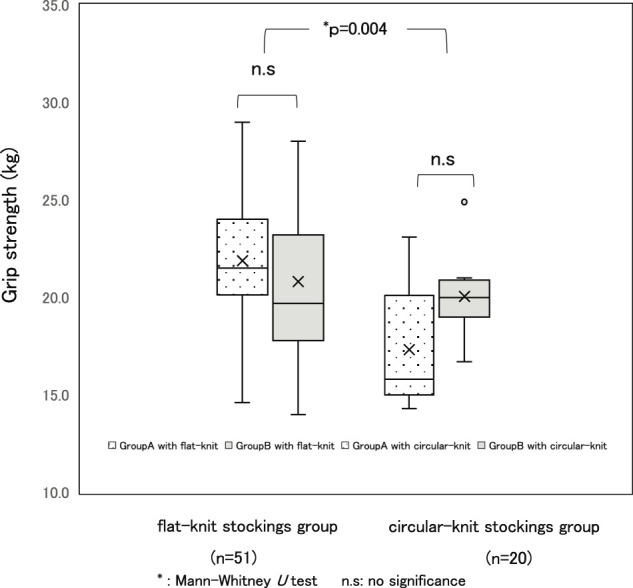
Comparison of grip strength between the flat-knitted compression stocking group and the circular-knit compression stocking group (n = 71).

We compared the grip strength of each group. Group A with flat-knit stocking group (n = 32) was 21.9 kg (range: 14.6–29.0kg) and that of Group B with flat-knit stocking group (n = 19) was 20.8 kg (range: 14.0–28 kg), with no significant difference between the 2 groups. Group A with circular-knit stocking group (n = 9) was 17.3 kg (range: 14.3–23.1kg) and that of Group B with circular-knit stocking group (n = 11) was 20.0 kg (range: 16.7–24.9 kg), with no significant difference between the 2 groups. Furthermore, we compared the grip strength of patients in the flat-knit stocking group (n = 51) and the circular-knit stocking group (n = 20). The grip strength of the flat-knit group was 21.1 kg (range: 14.0–29.0 kg), which was significantly higher than the 19.1 kg (range: 14.3–24.9 kg) of the circular-knit group (Mann–Whitney *U* test, *p* = 0.004).

## Discussion

Our findings demonstrate that older outpatients can maintain compression therapy effectively even into late-older stages. The patients were diagnosed as late-stage II according to the ISL classification. These patients are at high risk of worsening edema and developing cellulitis due to lymphatic dysfunction and require continued compression therapy with compression stockings.

Compression therapy using flat-knitted stockings with short-stretch is known to be effective in reducing edema in patients with late-stage II or later disease, as defined by the ISL classification, and this treatment is used by many patients.

Compression stockings used in lymphedema treatment are designed to provide higher compression pressure (Class II or above) than regular stockings: Class II stockings exert a pressure of approximately 30 mmHg on the ankle joint, making them difficult to don and doff. For patients with moderate-to-severe lymphedema, flat-knit stockings are highly effective in reducing edema; however, compared to circular-knit stockings, their fabric is stiffer and less elastic, and therefore, they are difficult to don and doff. A key clinical insight from this study is the association between grip strength and the types of compression stockings used. Although there was no difference in grip strength between the age groups, the grip strength of patients using flat-knit compression stockings was 2 kg greater than that of patients using circular-knit stockings, indicating that patients using flat-knit compression stockings require a certain level of grip strength.

The grip strength of Japanese women has been reported to be 25.1 ± 3.9 kg for those aged 65–69 years, 23.8 ± 4.0 kg for those aged 70–74 years, and 22.8 ± 4.0 kg for those aged 75–79 years; no data are available for those aged 80 years and older.^10)^ Furthermore, the age-specific physical fitness standards for grip strength stated in the Nursing Care Prevention Manual are 23.8 ± 4.0, 22.6 ± 3.9, 21.5 ± 3.7, and 19.6 ± 3.5 kg for those aged 65–69, 70–74, 75–79, and 80 years and older, respectively.^14)^ The grip strength of the patients in the current study tended to be slightly lower than the standard values. Despite relatively low grip strength, patients were able to continue compression therapy. Physical strength in older adults has improved in recent years; however, considerable individual variability remains.^15)^ In clinical practice, when a patient’s range of motion, dexterity, or grip strength declines—making it difficult to wear compression stockings, regardless of age—it may be necessary to consider switching from flat-knit to circular-knit compression stockings.

### Limitations

A limitation of this study was that grip strength was measured only once. Future studies should conduct regular assessments to monitor longitudinal changes in grip strength, activities of daily living, and disease progression. The number of clinics and participants in this study was limited; a larger sample size is needed for generalization.

## Conclusion

Even late-older patients could continue to use flat-knit stockings as long as their grip strength was maintained.
